# Is Anterior Cruciate Ligament Reconstruction “Silva Technique” Equal to All-Inside Techniques? A Prospective Single-Center Study: An Alternative ACL All-Inside Reconstruction Technique Using a Tibial Tunnel and Bone Graft

**DOI:** 10.1155/2024/2371242

**Published:** 2024-10-26

**Authors:** Panagiotis Karampinas, John Vlamis, Athanasios Galanis, Michail Vavourakis, Evangelos Sakellariou, Iordanis Varsamos, Ioannis Spyrou, Spiros Pneumaticos

**Affiliations:** 3rd Department of Orthopedic Surgery, National & Kapodistrian University of Athens, KAT General Hospital, Athens, Greece

**Keywords:** ACL, ACL reconstruction, all-inside techniques, anterior cruciate ligament, arthroscopy, bone graft, hamstrings, knee, tibial tunnel

## Abstract

**Background:** The development of less invasive all-inside techniques regarding anterior cruciate ligament (ACL) reconstruction surgery has been associated with various advantages, including fewer complications and reduced postoperative pain. Silva et al. described a quadruple semitendinosus graft construct and suspensory button fixation for ACL reconstruction as an alternative technique. At the end of this technique, the tibial tunnel is filled with a bone autograft plug. This paper aims to examine the incorporation of the autograft and thus evaluate whether the “Silva technique” provides the same benefits as all-inside techniques.

**Methods:** A prospective study assessed 31 patients undergoing ACL reconstruction surgery using the “Silva technique.” The cases involved in the study were skeletally mature patients with no previous history of ACL surgery or multiligamentous instability. All patients followed the same rehabilitation program and were examined at three standardized follow-up visits: 4 months, 8 months, and 1 year postoperatively. Tegner–Lysholm knee score (TLKS), visual analog score (VAS) for pain, and the IKDC subjective knee score were completed at every visit. A knee MRI scan was performed at every scheduled visit to assess bone graft incorporation and remodeling.

**Results:** TLKS scores revealed a considerable improvement compared to preoperative figures, from 57.2 points preoperatively to an average of 99.4 at the 12-month follow-up (*p* < 0.0001). VAS scores were substantially ameliorated after the operation and until the second follow-up visit, from 5 before surgery to zero 8 months after the operation, with no noteworthy alterations afterward (*p* < 0.0001). IKDC subjective knee score outcomes were found to have increased at the last follow-up, from 59.3 prior to surgery to 99.8 12 months postoperatively (*p* < 0.0001). Regarding the MRI features of the bone autograft, the tibial tunnel was entirely filled by bone formation at the last MRI scan, suggesting complete integration of the autograft in all patients.

**Conclusions:** Bone autograft employed to seal the tibial tunnel was completely incorporated in all cases 1 year postoperatively. The “Silva technique” appears to feature all the avails of all-inside techniques, whilst it seems to be simpler and easier than them after the surgeon is familiarized with its particular aspects. It is a robust option in orthopedic surgeons' arsenal. However, further large-scale pertinent research is requisite to confirm the findings of this study.

## 1. Introduction

Anterior cruciate ligament (ACL) reconstruction surgery is indubitably a well-established surgical procedure in orthopedics. The operation was first performed in the late nineteenth century, with Dr. Robson carrying out the first felicitous ACL reconstruction surgery [1, 2]. Over the years, owing to prodigious advancements in all aspects of knowledge and equipment, ACL reconstruction surgery techniques evolved to be considerably less invasive, specifically after the introduction of knee arthroscopy [3]. ACL reconstruction surgery was optimized to become less invasive, with markedly fewer complications, intraoperative bleeding, and postoperative pain [3].

One of the most significant advancements in ACL reconstruction surgery has been the development of the all-inside ACL reconstruction technique. This technique includes the formation of two closed-socket tunnels [4, 5], with double (femoral and tibial) suspensory fixation and smaller skin incisions [6]. Graft insertion is executed through an arthroscopic portal, minimizing postoperative bleeding, soft tissue damage, bone loss (abatement from 54% to 64%), as well as postoperative pain [7–9]. This technique might be a fruitful option when treating younger patients with open-growth plates to preserve and guarantee physiological skeletal growth [10]. Notwithstanding, in terms of all-inside ACL surgery, copious elements associated with this operation need to be clarified, including the appropriate timing of surgery, graft selection, graft fixation methods, particular operative techniques, and rehabilitation following the operation [11].

In 2015, Silva et al. delineated a quadruple semitendinosus graft construct and suspensory button fixation for ACL reconstruction as a promising alternative technique. In this technique, the semitendinosus graft construct is prepared in a typical quadruple method and fixed with the utilization of a cortical button in both femoral and tibial tunnels, with augmented stiffness and resistance of the graft making. At the end of the operation, the tibial tunnel is filled with a bone dowel. This procedure is considered safe, featuring a short learning curve while preserving the gracilis. It is considered more straightforward and accessible than the all-inside technique but features the same avails. As opposed to all-inside techniques, Silva's procedure requires a full tibial tunnel filled at the end of the surgery with a bone autograft plug, keeping the bone stock intact. Autografts have been described as having the main advantage of supplying bone volume and osteogenic cells capable of new bone formation through osteogenesis, osteoinduction, and osteoconduction [12]. Tibial fixation is carried out with the employment of a cortical button, a highly stiff and resistant fixation device, to avoid many of the problems ascribed to the biodegradable interference screw. Finally, the tibial bone plug prevents the enlargement of the tunnel [13]. Regarding the fixation of autografts, a recent randomized controlled trial by Mayr et al. contrasting tunnel widening and clinical results following ACL reconstruction surgery with interference screw fixation and all-inside reconstruction utilizing button fixation concluded that button fixation was correlated with less tibial tunnel widening and smaller tunnels 2 years postoperatively as opposed to screw fixation. Also, they reported that the requirement for staged revision ACL reconstruction surgery might be higher in terms of interference screws when contrasted with button fixation at the tibial tunnel, although clinical outcomes in both types of surgery were comparable [14]. Likewise, a more recent study by Eichinger et al. scrutinizing tunnel widening and clinical outcomes after ACL reconstruction surgery by comparing these two autograft fixation methods reported similar results.

Despite being vastly intriguing, the paper by Silva et al. describing this ACL reconstruction technique does have some limitations. The paramount aspect of this operation that needs to be sorted out is the efficacy of the tibial tunnel bone autograft placement. What remains vague is the tibial bone plug integration and the partial or even entire deformation. Thereby, the technique's capability in restoring tibial bone stock is still to be proven. Consequently, this paper aims to examine the incorporation and effectiveness of the tibial tunnel's bone graft plug, offering the same advantages as all-inside ACL reconstruction techniques while also evaluating the potency of Silva's technique. Also, our study aspires to distinguish and clarify typical MRI findings suggesting the incorporation of bone grafts. We hypothesized that the bone graft plug utilized in this technique would be fully incorporated in the majority of cases examined at 1 year postoperatively.

## 2. Materials and Methods

### 2.1. Design and Participants

In our orthopedics and trauma department, a prospective study was conducted evaluating ACL reconstruction surgeries employing the technique described by Silva and Sampaio [13] from October 2021 until March 2023. Our study was approved by the Hospital's Ethical and Scientific Committee. Thirty-one cases of ACL reconstruction surgery were included, 23 males and 8 females, with a mean age of 37 years (ranging from 28 to 45). Inclusion criteria were patients aged between 20 and 50 years old sustaining a first-occurred ACL rupture with negligible past medical history and no comorbidities. Furthermore, there were no associated knee injuries at the moment of surgery or during the follow-up period. Only skeletally mature patients were included, whilst patients with previous ACL reconstruction surgery or multiligamentous instability were excluded from the study. Also, in terms of exclusion criteria, patients with age outside of the limits of our study were excluded along with individuals with copious comorbidities receiving medication that could potentially affect the bone graft plug incorporation. The level of sports activity that the included patients were engaged in was sedentary to moderately active. The study initially involved 35 patients, however, only 31 individuals were finally included completing all the essential requirements of our study.

### 2.2. Surgical Procedure and Follow-Up

In all cases involved in our research, an ACL reconstruction operation was executed utilizing precisely the “Silva technique.” The same experienced surgical team carried out all operations. After surgery, all patients followed the same rehabilitation program of free range of motion from day one postoperatively, full weight-bearing and reinforcement exercises at 3 weeks postsurgery, while returning to light sports activities 8 weeks after the ACL operation. Regarding follow-up timeframes, all patients were assessed 4 months, 8 months, and 1 year postoperatively.

## 3. Procedures (Tests Performed)

The examining physician completed the Tegner–Lysholm knee score (TLKS), the visual analog score (VAS) for pain, and the IKDC subjective knee score for each patient during the scheduled follow-ups. Bone graft incorporation and remodeling were assessed with a knee MRI scan at the 4-month, 8-month, and first-year follow-up visit, respectively. The MRI was performed to investigate the presence or absence of marrow signal intensity, which is indicative of graft incorporation or failure, correspondingly [15, 16].

### 3.1. Statistical Analysis

Statistical analysis was carried out by employing Student's *t*-test for the continuous variables. In terms of statistical calculations, SPSS version 27.0 (IBM, New York, USA) was utilized. The statistical significance level was specified at *p* value < 0.05.

## 4. Results

In terms of subjective outcomes, TLKS scores demonstrated a considerable amelioration compared to preoperative values. The preoperative figures were 57.2 points, whilst, at the 12-month follow-up visit, postoperative scores upsurged pronouncedly to an average of 99.4 points (*p* < 0.0001). Regarding reported knee pain levels, as described by the patients' VAS pain scores, a noteworthy diminution was observed regarding self-reported pain levels, which were progressive throughout the 8th-month time-point, ranging from 5 prior to surgery to 2 at the 4-month follow-up visit, resulting in 0 after 8 months from the operation, after which no consequential alterations were detected (*p* < 0.0001). Concerning IKDC subjective knee score outcomes, they were found to increase from 59.3 before the ACL repair to 99.8 at the 12-month follow-up visit postoperatively (*p* < 0.0001) (Table 1).

On the other hand, by perusing the performed MRI scans, we recognized the distinctive characteristics of the tibial tunnel during the three follow-up periods. In general, autografts are characterized by variable appearances in MRI scans, as they might appear hyperintense on T1-weighted images and hypointense on T2-weighted images or hypointense on T1-weighted images and isointense to hyperintense on T2-weighted images [15].

**Figure 1:**
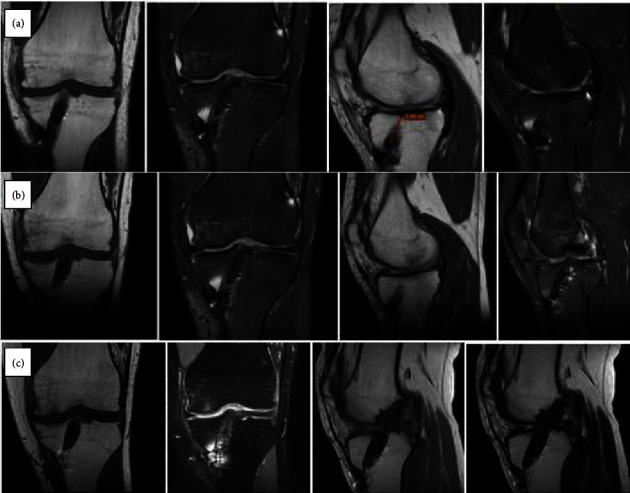
MRI graft images of a case during the 4th (a), 8th (b), and 12th (c) month follow-up.

**Figure 2:**
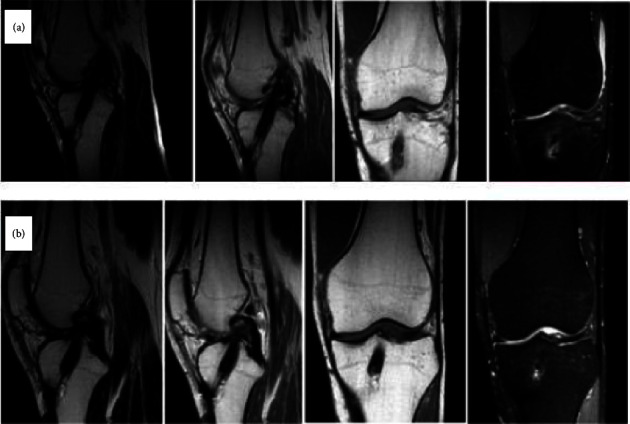
MRI graft images of a case during the 4th (a) and 12th (b) month follow-up.

At the first follow-up visit (4 months), the MRI characteristics of the bone autograft (Figures 1(a) and 2(a)) were identified in all cases. At the second follow-up (8 months), the same MRI bone autograft characteristics were recognized in all cases (Figures 1(b)), while the incorporation of the bone graft with a filling of the tibial tunnel was also detected. Finally, at the last follow-up visit (1-year postsurgery), we noticed that the tibial tunnel was entirely filled by the bone formation, and the ACL graft was stable in position (Figures 1(c) and 2(b)). Among all 31 patients evaluated, no notable discrepancies were observed regarding the MRI characteristics of the bone autograft.

## 5. Discussion

Arthroscopic ACL reconstruction is broadly considered a standardized surgical procedure. ACL reconstruction techniques are constantly developing as they become materially less invasive, preserving bone stock and tendons while providing better graft positioning and fixation [15, 17]. Transtibial drilling was the most ordinarily employed method for creating the femoral tunnel in conventional arthroscopic ACL reconstruction [18]. In this technique, the placement of the femoral socket is dictated by the tibial tunnel, leading to vertical graft placement. With the traditional transtibial technique, early outcomes were satisfactory. However, since the graft position is nonanatomical [19, 20], normal kinematics of the knee can be disturbed [21, 22], which could trigger persistent pain and early-onset knee arthritis [23].

Apart from the conventional transtibial technique, the transportal technique, where the femoral tunnel is drilled through an anteromedial portal, was developed to place the graft more anatomically, leading to meliorated knee stability in theory [16]. Transportal drilling of the femoral tunnel has been suggested to be associated with a more horizontal graft orientation of the ACL compared to transtibial drilling with no significant discrepancies in clinical outcomes; however, the transportal technique is correlated with a higher and earlier failure rate than the transtibial technique [12, 16, 24].

Contrariwise, the all-inside ACL reconstruction technique is widely regarded to feature copious advantages over the other techniques. The all-inside technique preserves gracilis, which can be utilized for potential future ligament repairs. The retained gracilis tendon maintains postoperative hamstring strength and serves as a significant secondary stabilizer [25]. The advantage of these procedures is the bone-sparing sockets that result in diminished postoperative pain, swifter postoperative recovery, and an essential convenience in case of possible ACL reconstruction revision [26]. Moreover, the all-inside technique enables precise graft positioning on the femoral and tibial side, which induces physiological advantages vital for promoting revascularization and ligamentization while also associated with enhanced knee flexor strength [27]. A contemporary systematic review by Bhimani et al. highlighted the superiority of all-inside techniques over the transportal technique in various aspects [26]. Moreover, all-inside ACL reconstruction techniques appear superior to complete tibial tunnel ACL reconstruction surgery in tibial tunnel widening and functional results. However, this was not the case regarding postoperative knee laxity and graft rerupture rates, where outcomes were similar [28]. Furthermore, a study by Genç et al. in 2023 examining the 6-month postoperative results of ACL reconstruction surgery with the modified all-inside (4ST) technique in athletes in terms of isokinetic strength values indicated very satisfying outcomes regarding knee strength regain and efficient return to sporting activities [29]. Likewise, the same year, a bigger-scale study by Mahirogullari et al. demonstrated that ACL reconstruction surgery incorporating the “modified all-inside” technique acquired considerable clinical outcomes contrasted to ACL reconstruction surgery with a suspensory femoral fixation and a bioabsorbable tibial interference screw [30]. Similarly, a 2023 retrospective cohort study examining 22 male recreational athletes who underwent modified all-inside ACL reconstruction surgery corroborated the efficacy of this technique when performed in athletes from a functional point of view [31]. Finally, a present-day study by Cerci et al. reported that patients undergoing ACL reconstruction surgery with quadrupled semitendinosus suspensory femoral and tibial fixation feature similar knee functionality and muscle strength compared to those individuals operated with four-strand semitendinosus-gracilis suspensory femoral fixation and a bioabsorbable tibial interference screw [32].

The technique described by Silva and Sampaio is considered safe, featuring a slight learning curve while preserving the gracilis, saving bone, and bolstering the stiffness and resistance of tibial fixation [13]. The chief points to evaluate in terms of Silva's ACL reconstruction technique are the following: Is the tibial bone graft plug incorporated? Is the tunnel sealed and refilled? If that is the case, are the typical advantages of all-inside techniques provided by the “Silva technique”? Our study intended to offer an answer to all these questions. At this point, it is imperative to underline that this paper aimed to provide comprehensive answers to these specific queries and not to describe the Silva's surgical procedure utilized during the operations of the study or to compare operative time and functional outcomes with other surgical techniques. For the exact surgical procedure in detail and for step-by-step surgical images it is advisable to refer to the original paper by Silva and Sampaio describing the technique in detail [13]. After 1-year follow-up of all cases involved in our research, the tibial bone graft plug was fully incorporated in all cases, and the tunnel was entirely sealed. In addition, the advantages of all-inside techniques, such as less postoperative pain, earlier full weight-bearing and quicker return to activities, and bone stock preserving, were present. Also, regarding the three scores used to evaluate pain and knee functionality, all outcomes significantly improved at the 1-year follow-up compared to the figures before ACL reconstruction surgery. Besides, in theory, the “Silva technique” might be more advantageous than the other ACL reconstruction techniques. More specifically, the anterior cruciate graft passes through the tibial tunnel without contact with the skin, thus potentially leading to less risk of postoperative infections. At the same time, it has been reported to be faster than the traditional technique [13]. In our study, operative time did not differ significantly in comparison with operations with the other ACL reconstruction techniques performed by the same experienced team. Notwithstanding, it is vitally important to accentuate that there is no study in the existing literature comparing Silva's technique with the others, thus a huge amount of pertinent research is required to evaluate whether this technique is indeed more advantageous than the others and to attain more solid inferences. With regards to our center, an interesting prospect for future research would be comparing Silva's technique with the all-inside ACL reconstruction technique, as the volume of all-inside ACL reconstruction surgery is steadily increasing in our center.

Reconstructive orthopedic procedures widely utilize bone graft materials to encourage new bone formation and healing. Both osteogenic cells and graft volume are supplied by an autograft, which will ultimately aid in bone formation. Osteogenesis, osteoinduction, and osteoconduction are three major processes that bone graft materials undergo and are responsible for new bone growth and formation. Osteoinduction is when mesenchymal cells from the surrounding tissue differentiate into osteoblasts. Osteoconduction takes place when an implant serves as a scaffold to expedite the ingrowth of vessels and the migration of host cells. As new bone is formed, the graft might be partially or utterly resorbed through a procedure delineated as creeping substitution [12, 15, 16].

Furthermore, mechanical stress facilitates the felicitous incorporation of bone grafts, contributing to new bone formation [15, 16]. Successful incorporation of bone graft material at any site depends on new bone formation, the graft's structural incorporation, and the skeleton's adaptive remodeling in response to mechanical stress. The imaging appearance of a bone graft depends on the type, composition, and age of the graft [12]. In our study, at the 1-year follow-up after the ACL reconstruction operation, MRI findings suggested that the bone autograft plug employed to seal the tibial tunnel was wholly incorporated, and new bone growth and formation were attained. The crucial role of the tibial bone dowel needs to be highlighted at this point for the successful execution of the Silva's technique and promising postoperative outcomes, however, further research is needed to consider and evaluate the potential complications associated with the tibial tunnel following ACL reconstruction surgery with this technique.

The dominant limitation of this study is the small number of patients examined. The explanation behind this fact is the comparatively high cost, as an MRI scan is the optimal tool to investigate bone graft integration course; nonetheless, the cost is too high and can be utilized in limited cases. At this point, we accentuate the requirement for further larger-scale pertinent research in this field, as our research could act as thought-provoking material.

## 6. Conclusion

We infer that the bone autograft utilized to seal the tibial tunnel during ACL reconstruction surgery employing the technique described by Silva et al. was entirely incorporated in all cases analyzed in the first year of follow-up. The “Silva technique” appears to maintain all the established advantages of all-inside ACL reconstruction techniques, plus an easier positioning of the ACL graft through the tibial tunnel without contact of the ACL graft to the skin. This paper considers this technique a viable option in the vast field of ACL reconstruction surgery after the orthopedic surgeon is familiarized with its particular aspects. Yet, considering our study's limitations, it is vitally important to underline the necessity for further apposite research to corroborate the findings.

## Figures and Tables

**Table 1:** Mean values of functional scores and pain VAS during follow-ups along with *p* values.

**Preop**		**p** **value**		**p** **value**		**p** **value**

IKDC	4MO	Preop/4 mo	8MO	Pre/8 mo	1 YR	Preop/12 mo
59.3	96.36	< 0.0001	99	< 0.0001	99.8	< 0.0001
TLKS						
57.2	97.2	< 0.0001	98.6	< 0.0001	99.4	< 0.0001
Pain VAS						
5	2	< 0.0001	0	< 0.0001	0	< 0.0001

## Data Availability

All raw data are available to access should they be requested.
